# Supplementary material for: Excess PrPC inhibits muscle cell differentiation via miRNA-enhanced liquid–liquid phase separation implicated in myopathy
Source: Nat Commun. 2023 Dec 8;14:8131. doi: 10.1038/s41467-023-43826-7 (PMC10709375; doi:10.1038/s41467-023-43826-7)
Supplement: Supplementary file 1 — Supplementary Information [file 41467_2023_43826_MOESM1_ESM.pdf]

Supplementary Information

Excess PrP^C inhibits muscle cell differentiation via miRNA-enhanced liquid–liquid phase separation implicated in myopathy

Jing Tao, Yanping Zeng, Bin Dai, Yin Liu, Xiaohan Pan, Li-Qiang Wang, Jie Chen, Yu Zhou, Zuneng Lu, Liwei Xie, Yi Liang

Supplementary Table

Supplementary Table 1 Clinical details of patients with dermatomyositis, neurogenic myopathy, and muscular dystrophy at the time when skeletal muscle samples were taken

Case	Gender	Age	Mean age ± S.D.	Clinical diagnosis
**Myopathy**				
1	F	52	50.9 ± 4.9	Dermatomyositis
2	F	49		Dermatomyositis
3	M	51		Neurogenic myopathy
4	F	58		Neurogenic myopathy
5	M	43		Muscular dystrophy
6	F	49		Muscular dystrophy
**Control**				
1	F	70	57.3 ± 17.1	Healthy individual
2	M	32		Lipid storage myopathy
3	M	64		Lipid storage myopathy
4	F	63		Glycogen storage disease

Here, the statistical test was not used for data analysis.

Supplementary Figures

Supplementary Figure 1

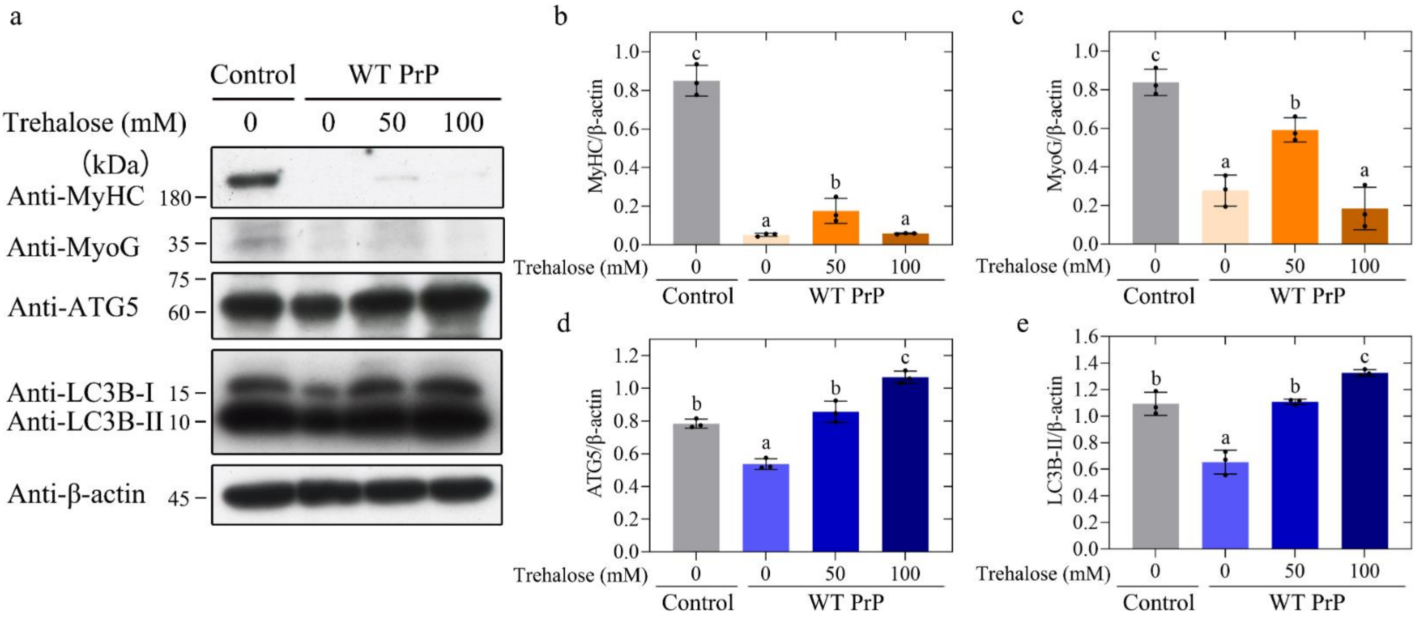

Supplementary Figure 1.

Autophagy is partially responsible for the effect of overexpression of PrP^{C} on myoblast differentiation.

a Western blot for PrP^{C}, the myogenic differentiation markers MyHC and MyoG, and the autophagy markers ATG5 and LC3B during C2C12 mouse myoblasts (control) and C2C12 myoblasts stably expressing full-length wild-type mouse PrP^{C} (WT PrP^{C}) cultured until their confluence reached 90% and then incubated with 50 or 100 mM trehalose and differentiation medium for 5 days. β-actin served as the protein loading control. **b–e** The relative amount of MyHC (**b**), MyoG (**c**), ATG5 (**d**), or LC3B-II (**e**) in the above cell lines (control, gray; WT PrP^{C}, 0 mM trehalose, light yellow (**b**, **c**) or light blue (**d**, **e**); WT PrP^{C}, 50 mM trehalose, orange (**b**, **c**) or blue (**d**, **e**); and WT PrP^{C},

100 mM trehalose, brown (**b**, **c**) or deep blue (**d**, **e**)) (solid black circles shown in scatter plots) was expressed as the mean ± SD (with error bars) of values obtained in $n = 3$ independent experiments. One-way two-sided ANOVA and multiple comparisons with no adjustments were performed by SPSS 19.0 and different letters indicate significant differences at the level of $p < 0.05$. Source data are provided as a Source Data file.

Supplementary Figure 2

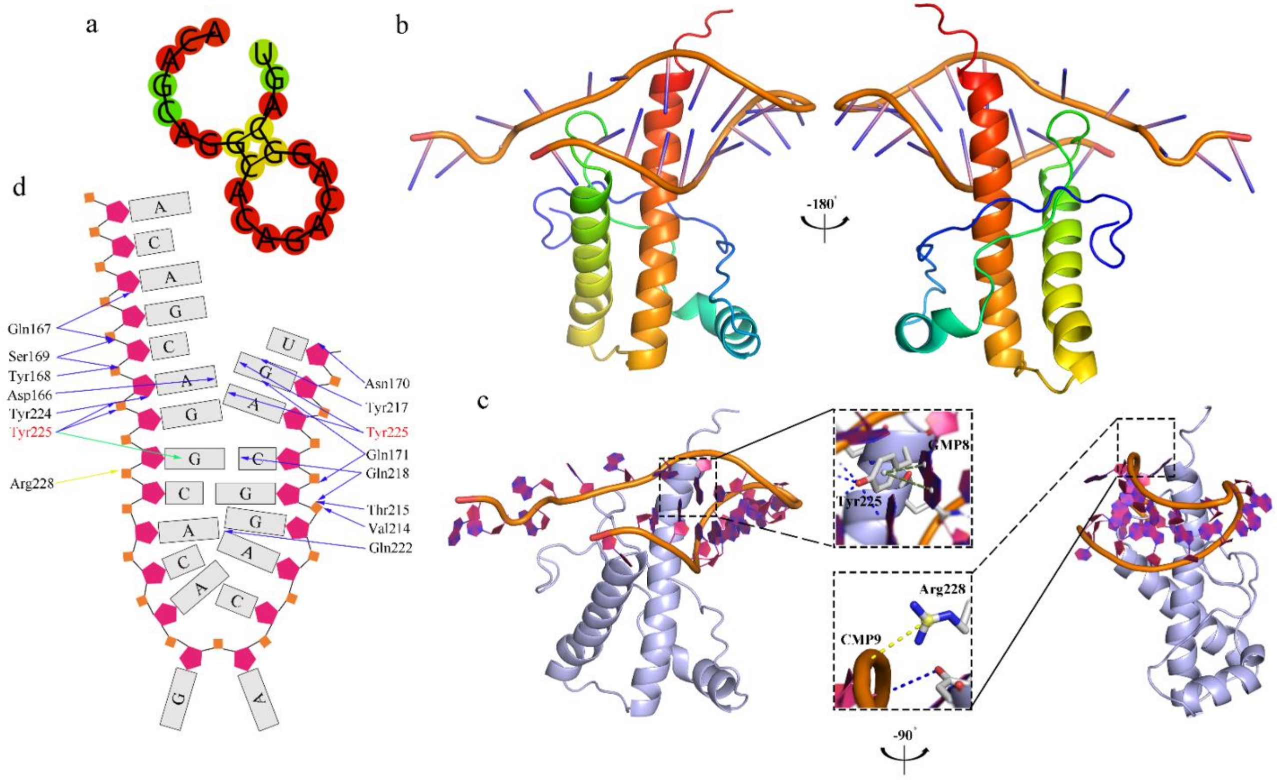

Supplementary Figure 2.

Prediction of the binding sites of miR-214-3p for PrP^{C}.

a Secondary structure of miR-214-3p with a key region highlighted in yellow (stem) predicted by RNAComposer. **b**, **c** Cartoon representation of the structure of the PrP^C:miR-214-3p complex in three different views. Molecular docking of PrP^C with miR-214-3p was performed using HDOCK. Ribbon representation of the structure of mouse PrP^C showing three α-helices (α1, α2, and α3) in the C-terminal domain of PrP^C (PDB 1XYX)[73]. **c** Predicting the positions of salt bridges, π-bonds, and hydrogen bonds using PyMOL. Two magnified side views (middle) of two regions of the interface between PrP^C and miR-214-3p highlighting two π-bonds between Tyr225 in PrP^C and GMP8 in miR-214-3p (green) and a salt bridge between Arg228 in PrP^C and CMP9 in miR-214-3p (yellow). Blue dashed lines represent abundant hydrogen bonds formed in the two regions of the interface. **d** Interactions between PrP^C and miR-214-3p. Asp166, Gln167, Tyr168, Ser169, Asn170, Gln171, Val214, Thr 215, Tyr217, Gln218, Gln222, Tyr224, and Tyr225 in PrP^C form abundant hydrogen bonds with two GCAG sequences present in miR-214-3p. Yellow, green, and blue arrows represent the observed salt bridge, π-bond, and hydrogen bonds formed in the complex, respectively.

Supplementary Figure 3

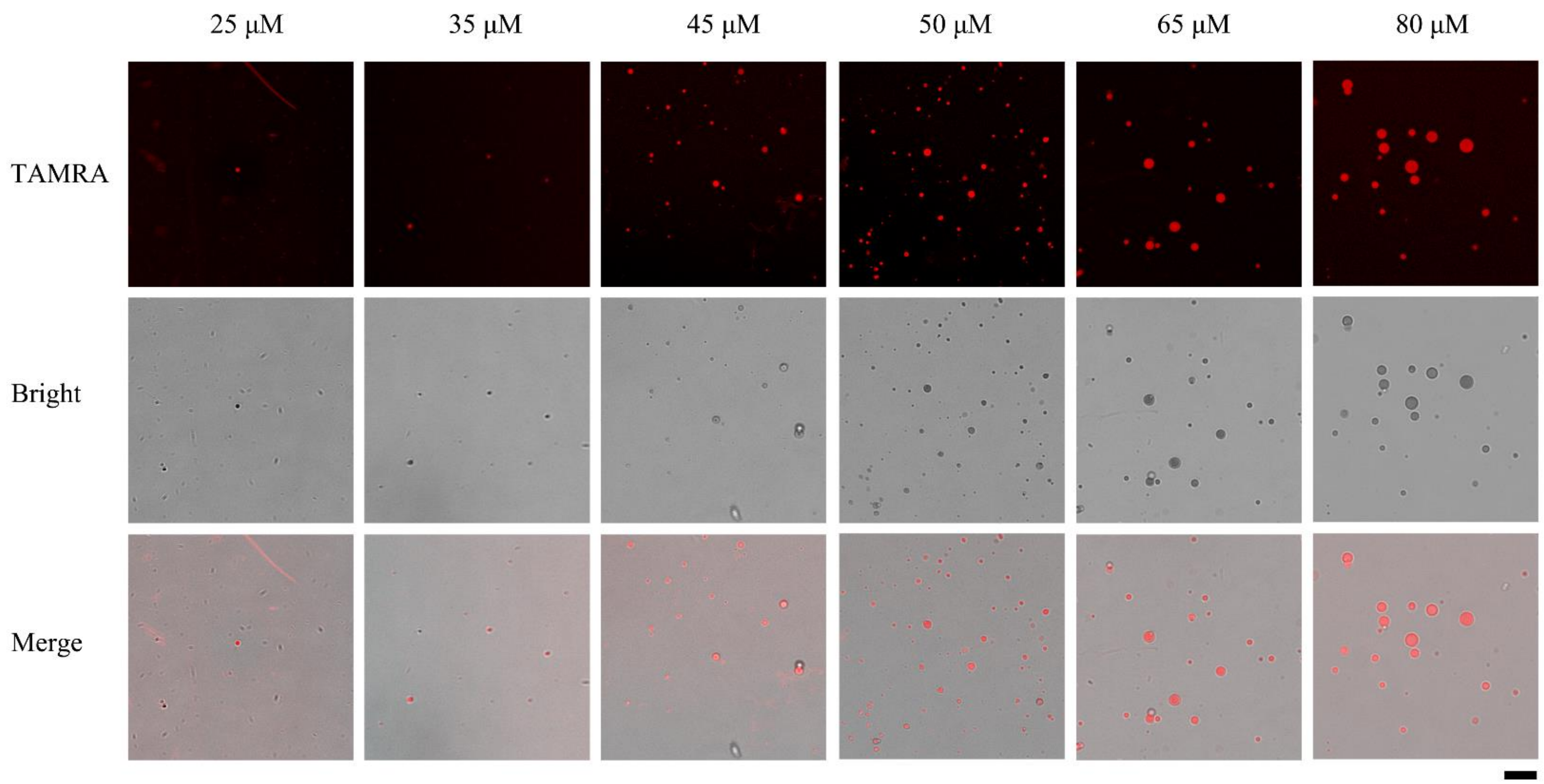

Supplementary Figure 3.

PrP^{C} undergoes liquid–liquid phase separation in vitro and forms protein condensates.

Samples (25, 35, 45, 50, 65, and 80 μM) of bacterial-purified wild-type mouse PrP^C were labeled by TAMRA (red) and incubated with 1 × PBS (pH 7.4) on ice to induce LLPS for 5 min. PrP^C de-mixed droplets (protein condensates) were observed by confocal microscopy, with excitation at 561 nm. The second and third rows show the brightfield images and the merged images for PrP^C LLPS, respectively. Scale bar, 7.5 nm. The experiments were repeated three times independently with similar results.
