# Supplementary material for: Excess PrPC inhibits muscle cell differentiation via miRNA-enhanced liquid–liquid phase separation implicated in myopathy
Source: Nat Commun. 2023 Dec 8;14:8131. doi: 10.1038/s41467-023-43826-7 (PMC10709375; doi:10.1038/s41467-023-43826-7)
Supplement: Supplementary file 3 — Description of Additional Supplementary Files [file 41467_2023_43826_MOESM3_ESM.pdf]

Description of Additional Supplementary Files

Supplementary Data Legends

Supplementary Data 1: Identification of 51 miRNAs bound by PrP^C in differentiating C2C12 cells stably expressing WT PrP^C versus 31 miRNAs bound by endogenous PrP^C in differentiating C2C12 cells (control).

Supplementary Data 2: Primers for vector constructions and small RNA oligos.

Supplementary Data 3: Primers for quantifying miRNA expression.

Supplementary Data 4: The FAM-labeled miRNAs, including FAM-labeled miR-214-3p, FAM-labeled miR-204-5p, FAM-labeled miR-83-5p, and FAM-labeled mutant miR-214-3p.

Supplementary Movie Legends

Supplementary Movie 1: Time-lapse imaging of C2C12 cells stably expressing mCherry-Cry2-WT PrP^C showing that two small liquid condensates gradually fused into one larger liquid droplet in living C2C12 cells.

Supplementary Movie 2: Time-lapse imaging of C2C12 cells stably expressing mCherry (red) upon transfection of FAM-labeled miRNA (green) showing that miRNA did not induce phase separation of mCherry in the aforementioned cells.

Supplementary Movie 3: Time-lapse imaging of C2C12 cells stably expressing mCherry (red) upon transfection of FAM-labeled miRNA (green) showing that miRNA did not induce phase separation of mCherry in the aforementioned cells.

Supplementary Movie 4: Time-lapse imaging of C2C12 cells stably expressing WT PrP^C-mCherry (red) upon transfection of FAM-labeled miRNA (green) showing that PrP^C demixed droplets (red; Merge: yellow) fused with droplets of miR-214-3p (green) and the colocalization of PrP^C and miR-214-3p (yellow puncta in the merged images) in phase-separated condensates.

Supplementary Movie 5: Time-lapse imaging of C2C12 cells stably expressing WT PrP^C-mCherry (red) upon transfection of FAM-labeled miRNA (green) showing that PrP^C demixed droplets (red; Merge: yellow) fused with droplets of miR-214-3p (green) and the colocalization of PrP^C and miR-214-3p (yellow puncta in the merged images) in phase-separated condensates.

Supplementary Movie 6: Time-lapse imaging of C2C12 cells stably expressing WT PrP^C-mCherry (red) showing that PrP^C puncta (red) exhibited features characteristic of liquid-like condensates.

Supplementary Movie 7: Time-lapse imaging of C2C12 cells stably expressing WT PrP^C-mCherry (red) upon transfection of miRNA showing that PrP^C and miR-214-3p puncta (red) exhibited features characteristic of liquid-like condensates.
