# Supplementary material for: Excess PrPC inhibits muscle cell differentiation via miRNA-enhanced liquid–liquid phase separation implicated in myopathy
Source: Nat Commun. 2023 Dec 8;14:8131. doi: 10.1038/s41467-023-43826-7 (PMC10709375; doi:10.1038/s41467-023-43826-7)
Supplement: Supplementary file 15 — Reporting Summary [file 41467_2023_43826_MOESM15_ESM.pdf]

nature portfolio

Corresponding author(s): Yi Liang

Last updated by author(s): Nov 7, 2023

Reporting Summary

Nature Portfolio wishes to improve the reproducibility of the work that we publish. This form provides structure for consistency and transparency in reporting. For further information on Nature Portfolio policies, see our Editorial Policies and the Editorial Policy Checklist.

Statistics

For all statistical analyses, confirm that the following items are present in the figure legend, table legend, main text, or Methods section.

n/a	Confirmed	
☐	☒	The exact sample size (*n*) for each experimental group/condition, given as a discrete number and unit of measurement
☐	☒	A statement on whether measurements were taken from distinct samples or whether the same sample was measured repeatedly
☐	☒	The statistical test(s) used AND whether they are one- or two-sided<br>*Only common tests should be described solely by name; describe more complex techniques in the Methods section.*
☒	☐	A description of all covariates tested
☒	☐	A description of any assumptions or corrections, such as tests of normality and adjustment for multiple comparisons
☐	☒	A full description of the statistical parameters including central tendency (e.g. means) or other basic estimates (e.g. regression coefficient) AND variation (e.g. standard deviation) or associated estimates of uncertainty (e.g. confidence intervals)
☐	☒	For null hypothesis testing, the test statistic (e.g. *F*, *t*, *r*) with confidence intervals, effect sizes, degrees of freedom and *P* value noted<br>*Give P values as exact values whenever suitable.*
☒	☐	For Bayesian analysis, information on the choice of priors and Markov chain Monte Carlo settings
☒	☐	For hierarchical and complex designs, identification of the appropriate level for tests and full reporting of outcomes
☒	☐	Estimates of effect sizes (e.g. Cohen's *d*, Pearson's *r*), indicating how they were calculated

Our web collection on statistics for biologists contains articles on many of the points above.

Software and code

Policy information about availability of computer code

Data collection	RIP-seq libraries were prepared using VAHTS Small RNA Library Prep Kit for Illumina.
Data analysis	The FASTQ file is encoded with Illumina version 1.8+, and the Q value of the base is obtained by subtracting the offset value of 33 from the ASCII value of all characters.The script independently developed by personal company was used to remove the adapter, and the quality of the sequence was cut according to the quality of the sequence. The original sequence was searched with a window of 5 bases in length. When the average sequencing quality of the bases in the window was lower than 20. Finally, the part starting from the front of the window will be truncated and discarded.Genome alignment analysis was performed using miRDeep2 software, in which the mapper.pl program called Bowtie to align Unique Reads with the reference genome sequence. The precursor and mature miRNA sequences of this species were downloaded from miRBase, and then the deduplicated sequences were aligned with them respectively to annotate the detected miRNAs.

For manuscripts utilizing custom algorithms or software that are central to the research but not yet described in published literature, software must be made available to editors and reviewers. We strongly encourage code deposition in a community repository (e.g. GitHub). See the Nature Portfolio guidelines for submitting code & software for further information.

Data

Policy information about availability of data

All manuscripts must include a data availability statement. This statement should provide the following information, where applicable:

- Accession codes, unique identifiers, or web links for publicly available datasets
- A description of any restrictions on data availability
- For clinical datasets or third party data, please ensure that the statement adheres to our policy

The RIP-seq data generated in this study have been deposited in the Gene Expression Omnibus (GEO) database under accession code GSE203419 [https://www.ncbi.nlm.nih.gov/geo/query/acc.cgi?acc=GSE203419]. The mouse genome (mm10, Genome Reference Consortium Mouse Build 38, GCA_000001635.2) was produced by the Mouse Genome Sequencing Consortium, and the National Center for Biotechnology Information (NCBI). The source data underlying Fig. 3a-d, Fig. 4c-e, Fig. 5b,f,g,j, Fig. 6, Fig. 7d, Fig. 8l-n, and Fig. 9a-e,h are provided as a Source Data.

Research involving human participants, their data, or biological material

Policy information about studies with human participants or human data. See also policy information about sex, gender (identity/presentation), and sexual orientation and race, ethnicity and racism.

Reporting on sex and gender	The study is not related to sex and gender, and sex and gender were not considered in study design.
Reporting on race, ethnicity, or other socially relevant groupings	The study is not related to race, ethnicity, or other socially relevant groupings, and the socially constructed or socially relevant categorization variable(s) were not used in our manuscript.
Population characteristics	Myopathy 1 (female, 52 years old, Dermatomyositis)<br>Myopathy 2 (female, 49 years old, Dermatomyositis)<br>Myopathy 3 (male, 51 years old, Neurogenic myopathy)<br>Myopathy 4 (female, 58 years old, Neurogenic myopathy)<br>Myopathy 5 (male, 43 years old, Muscular dystrophy)<br>Myopathy 6 (female, 49 years old, Muscular dystrophy)<br>Control 1 (female, 70 years old, Healthy individual)<br>Control 2 (male, 32 years old, Lipid storage myopathy)<br>Control 3 (male, 64 years old, Lipid storage myopathy)<br>Control 4 (female, 63 years old, Glycogen storage disease)
Recruitment	Recruitment was free of any potential self-selection bias or other bias that did not affect the results.
Ethics oversight	The study complies with all relevant ethical regulations. The study is based on analyses of skeletal muscle samples from two patients with dermatomyositis, two patients with neurogenic myopathy, and two patients with muscular dystrophy, and from one healthy individual, two patients with lipid storage myopathy, and one patient with glycogen storage disease (controls). Tissue materials were collected at the Department of Neurology, Renmin Hospital of Wuhan University after obtaining informed consent from the patients or their relatives, who did not receive any compensation. We have obtained consent to publish information that identifies individuals (including three or more indirect identifiers such as exact age, sex, and medical centre the study participants attended or rare diagnosis). All relevant regulations and legal requirements, including ethical approval from relevant authorities at Wuhan University, were observed during material collection. The biochemical work at Wuhan University was conducted based on a permission from the Wuhan University Ethics Committee (WAEF-2022-0073).

Note that full information on the approval of the study protocol must also be provided in the manuscript.

Field-specific reporting

Please select the one below that is the best fit for your research. If you are not sure, read the appropriate sections before making your selection.

☒ Life sciences ☐ Behavioural & social sciences ☐ Ecological, evolutionary & environmental sciences

For a reference copy of the document with all sections, see nature.com/documents/nr-reporting-summary-flat.pdf

Life sciences study design

All studies must disclose on these points even when the disclosure is negative.

Sample size	Three biologically independent samples were used for western blot assays (Fig. 3a, Fig. 4c, Fig. 6c,f, Fig. 8l, Fig. 9a,c, and Extended Data Fig. 1a), RIP-seq assays (Fig. 5a,c-e,h,i), qPCR assays (Fig. 5f,g and Fig. 6i-k), dual fluorescence report assays (Fig. 6a,b), and FRAP assays (Fig. 8i,j) with similar results obtained. Five biologically independent samples were used for the MTT assay (Fig. 9e) with similar results obtained. All aforementioned experiments were repeated three times or five times and the results were reproducible.
Data exclusions	No data were excluded from the analyses.

Replication	The western blot experiments were repeated three times with similar results to confirm the reproducibility. The RIP-seq experiments were repeated three times with similar results to confirm the reproducibility. The qPCR experiments were repeated three times with similar results to confirm the reproducibility. The dual fluorescence report experiments were repeated three times with similar results to confirm the reproducibility. The FRAP experiments were repeated three times with similar results to confirm the reproducibility. The MTT experiments were repeated five times with similar results to confirm the reproducibility. All Biochemical experiments in this paper were repeated independently from 3 to 5 times, and were all successfully reproduced.
Randomization	Our experiments were not related to randomization. Indicated concentrations of proteins and miRNAs were used for biochemical and cellular experiments.
Blinding	Blinding was not applied to our study. For the experiments in our paper, blinding methods were technically not possible: western blot assays, RIP assays, qPCR assays, dual fluorescence report assays, FRAP assays, and MTT assays. No animal studies were involved.

Reporting for specific materials, systems and methods

We require information from authors about some types of materials, experimental systems and methods used in many studies. Here, indicate whether each material, system or method listed is relevant to your study. If you are not sure if a list item applies to your research, read the appropriate section before selecting a response.

Materials & experimental systems

n/a	Involved in the study
☐	☒ Antibodies
☐	☒ Eukaryotic cell lines
☒	☐ Palaeontology and archaeology
☒	☐ Animals and other organisms
☒	☐ Clinical data
☒	☐ Dual use research of concern
☒	☐ Plants

Methods

n/a	Involved in the study
☒	☐ ChIP-seq
☒	☐ Flow cytometry
☒	☐ MRI-based neuroimaging

Antibodies

Antibodies used	Primary antibodies used are presented in the Methods section with validation referenced. They are:<br>anti-MyHC mouse monoclonal antibody (Developmental Studies Hybridoma Bank MAB4470-SP, 1:1,000 for immunoblotting),<br>anti-MyoG mouse monoclonal antibody (Santa Cruz Biotechnology, 1:500 for immunoblotting),<br>anti-8H4 mouse monoclonal antibody (Abcam, diluted 1:5,000 for immunoblotting),<br>anti-β-actin mouse monoclonal antibody (Beyotime AA128, 1:1,000 for immunoblotting),<br>anti-ATG5 rabbit monoclonal antibody (Sigma SAB5700062, 1:1,000 for immunoblotting),<br>anti-LC3B rabbit monoclonal antibody (Sigma SAB1306269, 1:1,000 for immunoblotting),<br>and anti-NCAM mouse monoclonal antibody ((Santa Cruz Biotechnology sc-106, 1:50 for immunohistochemistry)
Validation	anti-MyHC mouse monoclonal antibody against mouse MyHC in (Zhao et al. 2019. Cell Death and Disease 10, 183),<br>anti-MyoG mouse monoclonal antibody against mouse MyoG in (Wright, et al. 1989. Cell 56, 607-617),<br>anti-8H4 mouse monoclonal antibody validated against mouse PrP amino acids 145-180 in (Moreno et al. 2012 Nature 485, 507-511),<br>anti-β-actin mouse monoclonal antibody validated against actin in (Yamane et al. 2018 Nat Microbiol 4, 1096-1104),<br>anti-ATG5 mouse monoclonal antibody validated against ATG5 in (Dai. et al. 2021 J Biol Chem 297, 101222),<br>anti-LC3B mouse monoclonal antibody validated against LC3B in (Dai. et al. 2021 J Biol Chem 297, 101222),<br>and anti-NCAM mouse monoclonal antibody validated against NCAM in (Bourne et al. 1991 J Neurooncol 10, 111-119)

Eukaryotic cell lines

Policy information about cell lines and Sex and Gender in Research

Cell line source(s)	Murine-derived C2C12 myoblast cells (catalog number GDC0175) and HEK-293T cells (catalog number GDC0187) were obtained from China Center for Type Culture Collection (CCTCC, Wuhan, China).
Authentication	We verified the C2C12 cell line and the HEK-293T cell line according to the morphology by light microscopy.
Mycoplasma contamination	C2C12 cell line and HEK-293T cell line were negative in the mycoplasma contamination test.
Commonly misidentified lines (See ICLAC register)	No commonly misidentified lines were used in this study.

Plants

Seed stocks	The study is not related to plants and seed stocks.
Novel plant genotypes	The study is not related to plants and novel plant genotypes.
Authentication	The study is not related to plants, seed stocks, and novel plant genotypes. Therefore, authentication procedures for each seed stock used or novel genotype generated do not needed.
